# Alcohol Abstinence Is Associated with Regression of Non-Invasive Fibrosis Markers in Patients with Metabolic Syndrome: A 12-Month Prospective Study

**DOI:** 10.3390/jcm15062257

**Published:** 2026-03-16

**Authors:** Daniela Mihăilă, Horațiu-Paul Domnariu, Doru-Florian-Cornel Moga, Carmen-Daniela Domnariu

**Affiliations:** 1 Doctoral School of Medicine, Faculty of Medicine, “Lucian Blaga” University of Sibiu, 550024 Sibiu, Romania; 2 Department of Internal Medicine, “Dr. Alexandru Augustin” Military Clinical Emergency Hospital Sibiu, 550024 Sibiu, Romania; 3 Surgical Clinical Department, Faculty of Medicine, “Lucian Blaga” University of Sibiu, 550024 Sibiu, Romania; horatiupaul.domnariu@ulbsibiu.ro (H.-P.D.); cornel.moga@ulbsibiu.ro (D.-F.-C.M.); 4 Clinical Department of Surgery, “Dr. Alexandru Augustin” Military Clinical Emergency Hospital Sibiu, 550024 Sibiu, Romania; 5 Department of Dental Medicine and Nursing, Faculty of Medicine, “Lucian Blaga” University of Sibiu, 550024 Sibiu, Romania; carmen.domnariu@ulbsibiu.ro

**Keywords:** metabolic syndrome, alcohol consumption, liver fibrosis, non-invasive fibrosis markers, FIB-4, APRI, transient elastography

## Abstract

**Background:** Patients with metabolic syndrome represent a particularly vulnerable population for alcohol-related liver disease progression. However, real-world longitudinal data evaluating the impact of alcohol abstinence on liver fibrosis dynamics in this group remain limited. **Methods:** We conducted a prospective observational study including hospitalized adults with metabolic syndrome and chronic alcohol consumption. Clinical, laboratory, and non-invasive fibrosis markers—fibrosis-4 index (FIB-4), aspartate aminotransferase-to-platelet ratio index (APRI), and transient elastography—were assessed at baseline and after 6 and 12 months of individual follow-up. Patients were classified according to alcohol consumption status during follow-up. Longitudinal and comparative analyses were performed. **Results:** At baseline, patients were classified as having alcoholic steatosis (56.3%), alcoholic steatohepatitis (25.0%), or alcoholic cirrhosis (18.7%). During follow-up, 72.9% of patients achieved sustained alcohol abstinence. Abstinent patients demonstrated significant improvements in liver stiffness, FIB-4, and APRI scores at 12 months (all *p* < 0.001), while non-abstinent patients showed progressive worsening of fibrosis markers. Gamma-glutamyl transferase levels were independently associated with fibrosis severity at baseline. **Conclusions:** This prospective real-world study suggests that alcohol abstinence is associated with favorable longitudinal changes in non-invasive liver fibrosis markers in patients with metabolic syndrome. Given the non-invasive nature of the diagnostic approach and the relatively small sample size, these findings should be considered hypothesis-generating. Further studies with larger cohorts are warranted to better elucidate the interaction between metabolic risk factors, alcohol consumption, and liver disease progression.

## 1. Introduction

Metabolic syndrome is one of the most common conditions encountered in contemporary medical practice, with a continuously increasing global prevalence. Its main components abdominal obesity, insulin resistance, arterial hypertension, and dyslipidemia are closely associated with the development and progression of chronic liver diseases [1].

Liver involvement associated with metabolic syndrome follows a continuous spectrum of severity, beginning with simple hepatic steatosis and progressing to steatohepatitis; fibrosis; and, in advanced stages, cirrhosis. In this context, alcohol consumption, even at levels below the conventional thresholds used for diagnosing alcoholic liver disease, may exert additive or synergistic effects on hepatic injury in patients with metabolic syndrome [2]. Alcohol-related liver diseases, including alcoholic hepatic steatosis, alcoholic steatohepatitis, and alcoholic cirrhosis, are characterized by complex pathophysiological mechanisms such as oxidative stress, mitochondrial dysfunction, and activation of hepatic stellate cells [3].

The interaction between metabolic syndrome and alcohol consumption is particularly detrimental to liver health. Epidemiological evidence indicates that individuals who simultaneously present metabolic risk factors and alcohol consumption have a significantly increased risk of developing advanced liver fibrosis and liver-related complications compared with those presenting only one of these conditions [4,5]. Moreover, even moderate alcohol consumption may have clinically relevant hepatotoxic effects in patients with metabolic syndrome, in the absence of alcohol intake considered “abusive” according to traditional guidelines [6].

In clinical practice, early identification of liver fibrosis is essential for initiating therapeutic interventions and preventing progression to cirrhosis. In this regard, non-invasive methods for liver assessment have gained considerable importance over the past decade. Serological scores such as the fibrosis-4 index (FIB-4) and the aspartate aminotransferase-to-platelet ratio index (APRI), based on routinely available biochemical parameters (AST, ALT, and platelet count), as well as transient elastography (FibroScan), allow for the evaluation of liver fibrosis without the need for liver biopsy [7,8]. These tools are particularly useful for screening patients with multiple risk factors, such as those with metabolic syndrome and alcohol consumption.

Nevertheless, data regarding the evolution of liver involvement in relation to changes in alcohol consumption remain limited, and the impact of abstinence on outcomes has only recently been quantified in systematic syntheses [9].

Beyond lifestyle-related risk factors, several pharmacological agents have been investigated for their potential hepatoprotective effects across different etiologies of chronic liver disease, including viral hepatitis. These studies support the concept that shared, non–etiology-specific pathways—such as oxidative stress, inflammation, and fibrogenesis—may be modulated therapeutically, regardless of the underlying liver disease etiology [10]. However, despite these pharmacological advances, lifestyle interventions remain central to disease management, particularly in patients with metabolic syndrome and alcohol-related liver injury, for whom alcohol abstinence represents the most impactful and readily modifiable determinant of liver disease progression.

Therefore, the aim of the present study was to prospectively evaluate the impact of alcohol abstinence on the longitudinal evolution of non-invasive liver fibrosis markers, including FIB-4, APRI, and transient elastography, over a 12-month follow-up period in patients with metabolic syndrome and chronic alcohol consumption. Secondary objectives were to identify clinical and biochemical factors associated with baseline fibrosis severity and to compare fibrosis trajectories between abstinent and non-abstinent patients.

## 2. Materials and Methods

### 2.1. Study Design and Population

This prospective observational study included adult patients with metabolic syndrome and chronic alcohol consumption hospitalized in the Internal Medicine Department of the “Dr. Alexandru Augustin” Sibiu Military Emergency Clinical Hospital between 1 November 2024 and 31 October 2025.

The study was conducted in accordance with the principles of the Declaration of Helsinki. The study protocol was approved by the Ethics Committee of the “Dr. Alexandru Augustin” Military Emergency Clinical Hospital Sibiu on 31 October 2024 (approval no. 32, 31 October 2024), prior to the inclusion of the first study participant. The protocol was subsequently reviewed and approved for academic purposes by the Ethics Committee of the Faculty of Medicine, “Lucian Blaga” University of Sibiu on 6 May 2025 (approval no. 15, 6 May 2025).

All participants provided written informed consent prior to inclusion.

Metabolic syndrome was defined according to the National Cholesterol Education Program Adult Treatment Panel III (NCEP ATP III) criteria [11], requiring the presence of at least three of the following five components:

(1) abdominal obesity, defined as waist circumference > 102 cm in men or >88 cm in women; when waist circumference was not available, obesity was assessed using body mass index (BMI ≥ 30 kg/m^2^) as a surrogate marker;
(2) fasting plasma glucose ≥ 100 mg/dL or previously diagnosed type 2 diabetes mellitus;
(3) arterial hypertension (blood pressure ≥ 130/85 mmHg or current antihypertensive treatment);
(4) serum triglycerides ≥ 150 mg/dL or lipid-lowering treatment;
(5) reduced high-density lipoprotein cholesterol (HDL-C < 40 mg/dL in men or <50 mg/dL in women).

### 2.2. Inclusion and Exclusion Criteria

Inclusion criteria were: age ≥ 18 years; presence of metabolic syndrome as defined above; history of significant alcohol consumption documented by structured medical history and corroborated by biological markers (elevated gamma-glutamyl transferase and/or mean corpuscular volume); and written informed consent. Both day-hospitalized and continuously hospitalized patients were eligible.

Exclusion criteria included chronic viral hepatitis (HBV or HCV), autoimmune liver diseases, hepatocellular carcinoma, other chronic liver diseases of non-metabolic and non–alcohol-related etiology, acute or chronic alcoholic pancreatitis, and refusal to participate.

Alcoholic pancreatitis was excluded because it is frequently associated with acute or subacute systemic inflammation, metabolic disturbances, and alterations in liver-related laboratory parameters that may transiently influence serological fibrosis scores and liver stiffness measurements, independent of underlying hepatic fibrosis. Excluding patients with alcoholic pancreatitis allowed for a more accurate assessment of liver fibrosis dynamics attributable to metabolic syndrome and alcohol consumption.

### 2.3. Data Collection and Liver Assessment

Demographic, clinical, and anthropometric data were collected, including age, sex, area of residence, professional status, body mass index (BMI), alcohol consumption duration and quantity, smoking status, and metabolic comorbidities (hypertension, diabetes mellitus or impaired fasting glucose, dyslipidemia).

Laboratory assessments included complete blood count, liver enzymes (AST, ALT), gamma-glutamyl transferase (GGT), total bilirubin, albumin, international normalized ratio (INR), fasting glucose, and lipid profile.

Non-invasive liver fibrosis assessment was performed using the fibrosis-4 index (FIB-4), the aspartate aminotransferase-to-platelet ratio index (APRI), and transient elastography (FibroScan). Liver stiffness measurements were expressed in kilopascals (kPa), and fibrosis stages were defined according to validated cut-off values. For transient elastography, liver stiffness thresholds commonly used in alcohol-related liver disease were applied: <7.0 kPa for absent or mild fibrosis (F0–F1), 7.0–9.5 kPa for significant fibrosis (F ≥ 2), 9.5–12.5 kPa for advanced fibrosis (F ≥ 3), and ≥12.5 kPa for cirrhosis (F4).

All FibroScan examinations were performed by trained operator using standard procedures.

For correlation analyses, a composite variable defined as the presence of ≥2 metabolic comorbidities was used. This variable reflected the coexistence of at least two of the following conditions: arterial hypertension, diabetes mellitus or impaired fasting glucose, and dyslipidemia.

Significant alcohol consumption was defined as a history of chronic alcohol intake documented by structured medical history and corroborated by biological markers, including gamma-glutamyl transferase (GGT) levels above the upper limit of normal according to the institutional laboratory reference range (>55 U/L) and/or increased mean corpuscular volume (MCV > 100 fL), in the absence of alternative causes.

Alcohol intake was recorded by structured self-report, including beverage type, volume (mL), and consumption frequency (daily or weekly). For descriptive reporting, self-reported intake was converted to grams of ethanol per day using standard assumptions for alcohol content (beer 5%, wine 12%, spirits 40%) and ethanol density (0.789 g/mL). Entries lacking consumption frequency were not converted.

Patients identified as having alcohol dependence were systematically referred for psychiatric evaluation and specialized addiction therapy, in accordance with standard clinical practice. These interventions were part of routine care and were not standardized or evaluated as study-specific interventions.

Liver disease categories (alcoholic steatosis, alcoholic steatohepatitis, and alcoholic cirrhosis) were defined using a clinico-biological and imaging-based approach, reflecting routine real-world clinical practice. Liver biopsy was not systematically performed and was not required for study inclusion.

Alcoholic steatosis was defined by the presence of hepatic steatosis on imaging (ultrasound and/or transient elastography with controlled attenuation parameter when available), in the absence of clinical or laboratory signs suggestive of advanced liver disease.

Alcoholic steatohepatitis was defined by the presence of hepatic steatosis associated with persistent elevation of aminotransferases, biochemical markers of liver injury, and non-invasive fibrosis scores above steatosis ranges, without clinical features of cirrhosis.

Alcoholic cirrhosis was defined based on established clinical, laboratory, and elastographic criteria, including signs of portal hypertension and/or liver decompensation, compatible imaging findings, and liver stiffness values in the cirrhotic range.

Transient elastography was performed only when technically feasible (i.e., in the absence of clinically significant ascites). For patients who had ascites during hospitalization, elastography was performed after clinical control of ascites and when no free fluid was present at the measurement site, and only measurements meeting standard quality criteria were retained. Transient elastography measurements were considered reliable only when at least 10 valid measurements were obtained, with an interquartile range/median ratio ≤ 30% and a success rate ≥ 60%, in accordance with international recommendations.

Concomitant medications were recorded at baseline as part of routine clinical assessment, including antihypertensive agents, lipid-lowering therapy, antidiabetic medications, diuretics, and other drugs commonly prescribed in patients with metabolic syndrome and chronic liver disease. These treatments were continued according to standard of care and were not modified for study purposes.

### 2.4. Follow-Up and Group Allocation

Patients were evaluated at baseline, 6 months, and 12 months. Based on alcohol consumption status during follow-up, patients were classified into two groups: abstinent (complete cessation of alcohol consumption) and non-abstinent (continued alcohol consumption). Alcohol abstinence was defined as complete cessation of alcohol intake during follow-up, assessed by structured patient self-report and corroborated by biochemical markers (gamma-glutamyl transferase and mean corpuscular volume).

### 2.5. Statistical Analysis

Statistical analysis was performed using IBM SPSS Statistics version 26 (IBM Corp., Armonk, NY, USA). Continuous variables were tested for normality using the Shapiro–Wilk test and are presented as mean ± standard deviation or median (interquartile range), as appropriate. Categorical variables are expressed as frequencies and percentages.

Comparisons between groups were performed using Student’s *t*-test or Mann–Whitney U test for continuous variables and chi-square or Fisher’s exact test for categorical variables. Longitudinal changes were analyzed using repeated-measures ANOVA or the Friedman test, with Bonferroni correction for post hoc analyses.

Correlation analyses were conducted using Pearson or Spearman coefficients, as appropriate. Multivariate logistic regression was performed to identify independent predictors of liver fibrosis severity. A two-tailed *p*-value < 0.05 was considered statistically significant.

Due to its markedly right-skewed distribution, GGT was log-transformed prior to inclusion in regression analyses. A constant of 1 was added [log(1 + GGT)] as a standard numerical precaution, and not because zero values were present in the dataset.

## 3. Results

### 3.1. Baseline Characteristics of the Study Population

A total of 48 patients with metabolic syndrome and chronic alcohol consumption were included at baseline. According to the type of alcohol-related liver disease, patients were classified as alcoholic steatosis (*n* = 27), alcoholic steatohepatitis (*n* = 12), and alcoholic cirrhosis (*n* = 9). Baseline demographic, socio-professional, clinical, and laboratory characteristics are presented in Table 1.

Male sex predominated in the overall cohort (66.7%) and did not differ significantly across liver disease categories (*p* = 0.689). The mean age of the study population was 62.04 ± 9.78 years and was significantly higher in the cirrhosis subgroup compared to steatosis and steatohepatitis (*p* = 0.004). BMI was comparable across groups (*p* = 0.518). Most participants were from urban areas (54.2%), and 39.6% were retired. Current smoking was reported in 43.8% of patients.

Hypertension was the most frequent metabolic comorbidity (87.5%), followed by dyslipidemia (77.1%) and diabetes mellitus or impaired fasting glucose (50.0%), without significant differences between disease categories (Table 1).

At baseline, the median alcohol consumption in the overall cohort was 19.3 g ethanol/day (IQR 14.2–19.7), with significantly higher intake observed in patients with cirrhosis compared to those with steatosis or steatohepatitis (*p* < 0.001).

Among patients classified as having alcoholic cirrhosis, we recorded cirrhosis stage severity using Child–Pugh classification (A/B/C) and documented clinical decompensation features at baseline, including ascites and hepatic encephalopathy. 7 patients had ascites (mild/moderate–severe), and 4 had hepatic encephalopathy at baseline. 5 patients had a prior history of variceal bleeding and/or endoscopic evidence of portal hypertension.

Liver stiffness measurements were obtained only in patients in whom transient elastography was technically feasible and met quality criteria. In patients with clinically significant ascites at baseline, elastography was not performed at that time and fibrosis assessment relied on serological scores (FIB-4 and APRI) and clinical staging.

### 3.2. Correlations Between Clinical and Laboratory Parameters and Baseline Fibrosis Markers

Correlation analyses identified significant associations between baseline non-invasive fibrosis markers (FIB-4, APRI, and liver stiffness) and several clinical and laboratory variables, including age, aminotransferase levels, gamma-glutamyl transferase (GGT), platelet count, and the burden of metabolic comorbidities. Detailed correlation coefficients and significance levels are provided in Supplementary Table S1.

### 3.3. Baseline Fibrosis Markers According to Liver Disease Category

Baseline stratification of non-invasive fibrosis indices according to liver disease category revealed significant differences across groups. Patients with alcoholic cirrhosis had substantially higher FIB-4, APRI, and liver stiffness values compared with those with steatosis or steatohepatitis (all *p* < 0.001). The proportion of patients with significant and advanced fibrosis increased progressively with disease severity. Detailed baseline stratifications are presented in Supplementary Table S2.

### 3.4. Follow-Up and Alcohol Abstinence Status

During follow-up, 35 patients (72.9%) achieved sustained alcohol abstinence, whereas 13 patients (27.1%) continued alcohol consumption. All patients completed the scheduled 6- and 12-month follow-up evaluations, with no loss to follow-up. At both follow-up time points, non-abstinent patients were older, more frequently had cirrhosis, and exhibited higher non-invasive fibrosis marker values compared with abstinent patients. Comparative analyses at individual follow-up visits are detailed in Supplementary Table S3.

### 3.5. Longitudinal Evolution of Non-Invasive Fibrosis Markers

**Figure 1:**
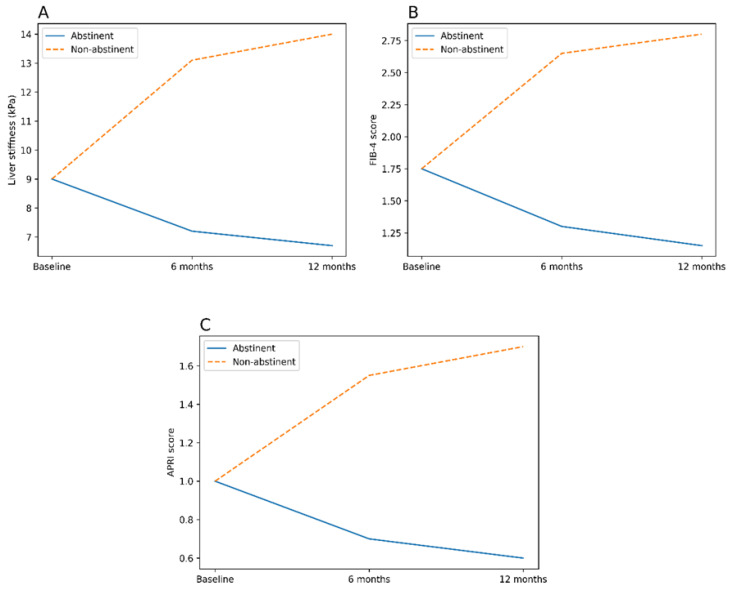
Longitudinal evolution of non-invasive liver fibrosis markers according to alcohol abstinence status: (**A**) liver stiffness assessed by transient elastography (FibroScan), (**B**) fibrosis-4 index (FIB-4), and (**C**) Aspartate Aminotransferase-to-Platelet Ratio Index (APRI). Solid lines represent abstinent patients, whereas dashed lines represent non-abstinent patients.

The longitudinal evolution of fibrosis markers according to alcohol abstinence status is summarized in Table 2 and illustrated in Figure 1A–C. Among abstinent patients, significant and sustained reductions in GGT levels, FIB-4, APRI, and liver stiffness were observed at 6 months and persisted at 12 months (all *p* < 0.001). In contrast, patients who continued alcohol consumption exhibited persistently elevated and progressively worsening fibrosis markers over time. These divergent trajectories highlight a strong association between sustained alcohol abstinence and favorable longitudinal changes in non-invasive fibrosis markers in patients with metabolic syndrome.

Additional detailed analyses of liver disease distribution and longitudinal changes in clinical and fibrosis markers at 12 months are provided in the Supplementary Materials (Tables S3 and S4).

### 3.6. Multivariate Analysis

In multivariate regression analysis, log-transformed gamma-glutamyl transferase emerged as the only independent predictor of baseline fibrosis severity across different non-invasive assessment methods. No other demographic, metabolic, or clinical variables remained independently associated after adjustment. Detailed regression results are reported in Supplementary Table S4.

## 4. Discussion

This prospective observational study provides robust real-world evidence regarding the complex interaction between metabolic syndrome and alcohol consumption in determining liver fibrosis severity and its longitudinal evolution. The principal finding of this study is that alcohol abstinence is associated with significant and sustained improvement in non-invasive liver fibrosis markers, whereas continued alcohol consumption is linked to fibrosis progression over a 12-month follow-up period in patients with metabolic syndrome.

Although the laboratory parameters used in this study are routinely available, their longitudinal integration into non-invasive fibrosis scores and elastographic assessment provides clinically meaningful insights into fibrosis dynamics. The observed concordant trajectories of FIB-4, APRI, and liver stiffness according to alcohol abstinence status underscore the utility of these accessible tools for monitoring disease evolution in real-world patients with metabolic syndrome and alcohol-related liver disease.

### 4.1. Interaction Between Metabolic Syndrome and Alcohol-Related Liver Injury

Metabolic syndrome amplifies hepatic vulnerability to alcohol-related injury through synergistic mechanisms involving insulin resistance, oxidative stress, and chronic low-grade inflammation. Our findings provide longitudinal real-world evidence supporting the emerging MetALD paradigm, demonstrating that patients with metabolic syndrome experience rapid fibrosis progression in the absence of alcohol abstinence. Importantly, even moderate or ongoing alcohol consumption was associated with worsening non-invasive fibrosis markers, highlighting that traditional “safe” alcohol thresholds may not apply to this metabolically vulnerable population.

At baseline, we observed a clear gradient of fibrosis severity across alcohol-related liver disease categories, with progressively higher FIB-4, APRI, and FibroScan liver stiffness values from steatosis to cirrhosis. These findings are consistent with recent evidence indicating that metabolic dysfunction acts as a major modifier of alcohol-related liver injury, amplifying fibrosis progression and disease severity when both conditions coexist [12,13,14,15]. Recent population-level analyses, cohort studies, and expert consensus published between 2023 and 2025 indicate that metabolic dysfunction substantially modifies the hepatic response to alcohol exposure, increasing susceptibility to hepatocellular injury and fibrosis progression when metabolic and alcohol-related risk factors coexist, including at alcohol intake below conventional alcohol-associated liver disease thresholds [16,17,18].

Recent consensus articles have defined the MetALD concept and highlighted the synergistic effects of metabolic dysfunction and alcohol exposure on liver injury and progression to fibrosis [19,20]. In this context, our findings provide prospective real-world evidence supporting the clinical relevance of this interaction, demonstrating divergent fibrosis trajectories according to alcohol abstinence status.

From a pathophysiological perspective, the coexistence of insulin resistance, visceral adiposity, and dyslipidemia promotes hepatic lipid accumulation and lipotoxicity, triggering hepatocellular stress responses and chronic low-grade inflammation. These mechanisms can potentiate alcohol-related oxidative stress and mitochondrial dysfunction and, downstream, favor a profibrogenic milieu with hepatic stellate cell activation and extracellular matrix remodeling [21,22,23]. Recent mechanistic studies have highlighted shared molecular pathways—such as dysregulated lipid metabolism, endoplasmic reticulum stress, and pro-inflammatory cytokine signaling—that contribute synergistically to fibrosis progression in patients with combined metabolic and alcohol-related risk factors [24,25].

Importantly, accumulating evidence suggests that individuals with metabolic syndrome or metabolic dysfunction are more susceptible to alcohol-related liver injury and fibrosis progression, such that alcohol exposure levels considered low or moderate in metabolically healthy populations may be associated with advanced liver disease in this setting [2,6].

It should be acknowledged that changes in non-invasive fibrosis markers, particularly liver stiffness measured by transient elastography, may be influenced by factors other than true fibrosis regression. Previous studies have demonstrated that alcohol consumption and, conversely, alcohol withdrawal can lead to rapid changes in liver stiffness, reflecting improvements in hepatic inflammation, hepatocellular ballooning, and portal pressure rather than structural remodeling of fibrosis.

This distinction is particularly relevant in the interpretation of longitudinal elastography data in alcohol-related liver disease. In a landmark study, Gelsi et al. showed a marked decrease in liver stiffness shortly after alcohol detoxification, suggesting that early elastographic changes primarily reflect resolution of alcohol-related inflammation rather than histological fibrosis regression [26]. Similar observations have been reported in subsequent studies evaluating transient elastography dynamics in alcohol-related liver disease.

In this context, the longitudinal improvements observed in our abstinent patients—particularly within the first 6 months—should be interpreted as reflecting a combination of reduced necroinflammatory activity and, potentially, early fibrosis regression. Importantly, the persistence of improvements at 12 months, together with concordant reductions in serological fibrosis indices (FIB-4 and APRI), supports the clinical relevance and consistency of sustained alcohol abstinence–associated improvements, while acknowledging the limitations of non-invasive markers in disentangling inflammation from fibrosis regression.

### 4.2. Alcohol Abstinence and Fibrosis Regression

A key finding of the present study is the significant improvement in non-invasive fibrosis markers among patients who achieved alcohol abstinence. Reductions in FIB-4, APRI, and FibroScan liver stiffness were observed as early as 6 months and were maintained at 12 months. In contrast, patients who continued alcohol consumption exhibited persistently elevated and progressively worsening fibrosis markers.

These findings are concordant with longitudinal studies and meta-analyses demonstrating that alcohol abstinence is the most effective modifiable factor for improving liver-related outcomes in alcohol-related liver disease [27,28,29]. FibroScan-based studies have further shown that liver stiffness may decrease rapidly following alcohol cessation, reflecting improvements in necroinflammation and portal pressure, with potential early fibrosis regression [30,31].

Our results extend this evidence by focusing specifically on patients with metabolic syndrome, a subgroup increasingly recognized as particularly vulnerable to alcohol-induced liver injury. Despite the presence of multiple metabolic comorbidities, abstinent patients in our cohort demonstrated meaningful improvements in fibrosis markers, highlighting the potential reversibility of liver damage when alcohol exposure is eliminated.

### 4.3. Utility of Non-Invasive Fibrosis Markers in Longitudinal Assessment

Importantly, while the deleterious effects of alcohol on liver fibrosis are well established, the novelty of the present study lies in its prospective, real-world evaluation of fibrosis dynamics in patients with metabolic syndrome, a population increasingly recognized as particularly vulnerable to alcohol-related liver injury. By integrating longitudinal changes across three independent non-invasive fibrosis assessment tools, our findings provide practical insights into disease monitoring beyond static baseline risk stratification and reflect real-life clinical trajectories rather than controlled trial conditions.

The consistent findings observed across FIB-4, APRI, and FibroScan underscore the clinical utility of non-invasive fibrosis assessment for monitoring disease evolution and therapeutic response. Recent international guidelines and consensus statements increasingly recommend the combined use of serological scores and elastography for risk stratification and follow-up in patients with metabolic-associated and alcohol-related liver disease [32].

Several recent studies have validated the prognostic value of these tools for predicting liver-related events and mortality, particularly when applied longitudinally rather than as single time-point assessments [33,34]. Our data support the integration of these non-invasive methods into routine clinical practice, especially for patients with overlapping metabolic and alcohol-related risk factors.

### 4.4. Determinants of Fibrosis Severity and the Role of GGT

In multivariate analysis, gamma-glutamyl transferase (GGT) emerged as the only independent predictor of fibrosis severity across different non-invasive assessment methods. This finding aligns with accumulating evidence identifying GGT as a biomarker that integrates alcohol-related oxidative stress and metabolic dysfunction [35,36].

Recent studies have demonstrated that elevated GGT levels are associated with increased hepatic fibrogenesis, cardiovascular risk, and liver-related mortality, independent of traditional liver enzymes [37]. In the context of metabolic syndrome, GGT may reflect increased hepatic fat content, insulin resistance, and oxidative stress, thereby serving as a sensitive marker of combined metabolic and alcohol-related liver injury [35].

The lack of independent associations for age, BMI, and platelet count after adjustment may reflect the dominant pathogenic role of alcohol-induced oxidative stress in this cohort, particularly when compounded by metabolic dysfunction.

### 4.5. Shared Molecular Mechanisms Between Alcohol-Related Liver Disease and MASLD

While our findings demonstrate improvements in non-invasive fibrosis markers following alcohol abstinence, the underlying biological mechanisms linking alcohol exposure and metabolic liver injury warrant further consideration.

A growing body of evidence suggests that alcohol does not act solely as an additive hepatotoxic factor in patients with metabolic syndrome but rather as an independent amplifier of the molecular circuits that also drive metabolic dysfunction–associated steatotic liver disease (MASLD). Recent molecular and metabolic studies demonstrate that alcohol-related liver disease and MASLD share several downstream pathogenic pathways, including oxidative stress, mitochondrial dysfunction, activation of inflammatory signaling cascades such as NF-κB, increased de novo lipogenesis, and hepatic stellate cell activation leading to fibrogenesis. In this context, alcohol exposure may exacerbate hepatic injury by intensifying the same lipotoxic, inflammatory, and fibrogenic mechanisms already activated by metabolic dysregulation. Consequently, alcohol abstinence may provide hepatic benefit not only through the removal of a direct hepatotoxic insult but also by simultaneously reducing oxidative, inflammatory, and lipogenic stress within overlapping pathogenic pathways. This concept of mechanistic convergence offers a plausible biological explanation for the improvements observed in non-invasive markers of liver fibrosis in our study population, including reductions in APRI and FIB-4 scores and improvements in elastographic parameters. Even in individuals with persistent metabolic risk factors, removal of alcohol exposure may reduce the burden on shared molecular injury pathways, thereby attenuating hepatic inflammation and fibrogenesis. These observations align with recent studies highlighting the shared molecular framework between alcohol-associated liver disease and MASLD, which indicate that alcohol acts as an independent amplifier of metabolic liver injury, whereas abstinence reduces the burden on convergent oxidative and inflammatory pathways involved in both conditions [38,39].

These findings support the concept that alcohol abstinence may alleviate hepatic injury not only by eliminating a direct hepatotoxic exposure but also by reducing the burden on shared metabolic and inflammatory pathways involved in both alcohol-associated and metabolic liver disease.

### 4.6. Clinical and Public Health Implications

From a clinical perspective, these findings emphasize the importance of systematic screening for liver fibrosis in patients with metabolic syndrome who consume alcohol, regardless of the reported quantity of intake. Non-invasive tools such as FIB-4, APRI, and transient elastography (FibroScan) are readily available, cost-effective, and suitable for longitudinal monitoring, supporting their routine use in clinical practice for risk stratification and follow-up in high-risk populations [40,41]. Moreover, our results indicate that alcohol abstinence should be regarded as a cornerstone intervention in this vulnerable population, irrespective of baseline fibrosis stage. The observed improvements across three independent non-invasive fibrosis assessment tools within a 6–12-month timeframe underscore the value of repeated fibrosis assessment, structured abstinence counseling, and comprehensive metabolic risk factor control, including weight reduction, glycemic optimization, and lipid management. Recent international guidelines and expert consensus statements increasingly advocate for integrated management strategies addressing both metabolic dysfunction and alcohol exposure [42].

At a public health level, our findings reinforce the growing consensus that no level of alcohol consumption can be considered safe in individuals with metabolic syndrome or metabolic dysfunction–associated liver disease. Rather than supporting the concept of “safe” alcohol thresholds, our results align with contemporary evidence indicating that metabolic vulnerability amplifies the hepatotoxic effects of alcohol, even at low levels of intake, supporting calls for strict alcohol avoidance in metabolically vulnerable populations [16]. Furthermore, alcohol consumption remains a major public health concern in Romania, where it has been associated with increased premature mortality, underscoring the importance of early identification, prevention strategies, and targeted interventions aimed at reducing alcohol-related morbidity and mortality [43,44].

While the present study did not include a formal health economic analysis, the observed association between alcohol abstinence and improved fibrosis trajectories may have potential downstream economic implications, by reducing the risk of cirrhosis progression, liver-related complications, and healthcare utilization. These aspects warrant dedicated health economic evaluation in future studies.

### 4.7. Strengths and Limitations

The strengths of this study include its prospective design, real-world clinical setting, longitudinal follow-up, and comprehensive assessment using multiple validated non-invasive fibrosis markers. The concordance of findings across different assessment methods strengthens the robustness of the conclusions.

However, several limitations should be acknowledged. The relatively small sample size, particularly in the cirrhosis subgroup, may have limited the power to identify independent predictors of advanced fibrosis. Alcohol consumption was assessed primarily through self-report and routine biochemical markers, which may be subject to reporting bias. Additionally, dietary intake and physical activity were not systematically controlled and may have influenced fibrosis dynamics independently of alcohol consumption.

The use of BMI instead of waist circumference for defining obesity may have led to misclassification of central adiposity in some patients.

The predominance of intermediate to advanced stages among patients with cirrhosis in the present cohort likely reflects the hospital-based, real-world design of the study. Patients with compensated cirrhosis are often managed in outpatient settings and are therefore underrepresented among hospitalized populations. In addition, individuals with metabolic syndrome and chronic alcohol consumption may remain asymptomatic for prolonged periods, which can lead to delayed diagnosis and presentation at more advanced stages, particularly in the absence of systematic fibrosis screening. These factors should be considered when interpreting the stage distribution of cirrhosis in our study.

Although strict alcohol abstinence was associated with favorable fibrosis trajectories, it should be noted that patients with alcohol dependence were recommended psychiatric consultation and specialized addiction therapy as part of routine clinical care. However, the present study did not assess the effectiveness, adherence, or specific modalities of these interventions. Therefore, our findings support the clinical importance of abstinence, rather than the efficacy of any particular therapeutic strategy.

Finally, liver fibrosis and disease categories were assessed exclusively using non-invasive methods, without systematic histological confirmation, and the observational nature of the study precludes causal inference. The absence of liver biopsy limits histological confirmation of steatohepatitis; however, the applied clinico-paraclinical classification reflects real-world clinical practice and aligns with current guideline-supported non-invasive approaches. Accordingly, residual confounding by unmeasured or incompletely measured factors cannot be fully excluded, and subgroup analyses should be interpreted with caution. Therefore, the findings should be considered hypothesis-generating rather than definitive.

Importantly, although the sample size was relatively small, the prospective design, complete follow-up, and concordant findings across three independent non-invasive fibrosis assessment tools enhance the robustness and internal validity of the results.

Although concomitant medications were recorded, their potential effects on non-invasive fibrosis markers were not analyzed separately, which may represent a source of residual confounding inherent to the real-world observational design.

### 4.8. Future Research Directions

Future research should prioritize targeted interventional and implementation-oriented studies rather than additional large-scale observational cohorts. In particular, pragmatic trials evaluating structured alcohol cessation programs integrated with metabolic risk factor management may provide clinically meaningful insights into optimizing care for patients with metabolic syndrome and alcohol-related liver disease. The integration of objective alcohol biomarkers and standardized lifestyle assessments could further refine exposure characterization and improve real-world applicability of guideline-recommended strategies [16,45].

## 5. Conclusions

In this prospective observational study, alcohol abstinence was associated with favorable longitudinal changes in multiple non-invasive liver fibrosis markers in patients with metabolic syndrome and chronic alcohol consumption. In contrast, continued alcohol intake was associated with worsening fibrosis trajectories, supporting the concept that metabolic dysfunction amplifies susceptibility to alcohol-related liver injury.

From a clinical perspective, these findings underscore the importance of active liver fibrosis screening and longitudinal monitoring in patients with metabolic syndrome who consume alcohol, even in the absence of overt alcohol abuse or advanced liver disease at baseline. The consistent longitudinal behavior of routinely available non-invasive tools (FIB-4, APRI, and transient elastography) supports their practical use for risk stratification, follow-up, and counseling in this metabolically vulnerable population.

However, given the observational design of the study, the results should be interpreted as associative rather than causal and should not be construed as clinical practice recommendations. Rather than calling for additional large-scale observational studies, future research should focus on targeted interventional and implementation-oriented approaches aimed at optimizing fibrosis monitoring strategies and alcohol cessation interventions in patients with metabolic syndrome. Specifically, pragmatic studies integrating structured abstinence programs with metabolic risk factor management may provide added clinical value beyond existing guideline recommendations.

## Figures and Tables

**Table 1:** Baseline demographic, socio-professional, clinical and laboratory characteristics of the study population according to type and severity of alcohol-related liver disease.

Characteristic	Total (*n* = 48)	Steatosis (*n* = 27)	Steatohepatitis (*n* = 12)	Cirrhosis (*n* = 9)	*p*-Value	Child-Pugh A (*n* = 2)	Child–Pugh B (*n* = 4)	Child–Pugh C (*n* = 3)
Sex								
Male, *n* (%)	32 (66.7)	17 (63.0)	8 (66.7)	7 (77.8)	0.689	1 (50.0)	3 (75.0)	3 (100.0)
Female, *n* (%)	16 (33.3)	10 (37.0)	4 (33.3)	2 (22.2)	–	1 (50.0)	1 (25.0)	0 (0.0)
Age (years)								
Mean ± SD	62.0 ± 9.8	58.9 ± 8.4	61.8 ± 8.0	69.1 ± 6.4	0.004	66.5 ± 5.1	68.2 ± 6.0	73.9 ± 4.8
Environment of origin								
Urban, *n* (%)	26 (54.2)	16 (59.3)	6 (50.0)	4 (44.4)	0.712	1 (50.0)	2 (50.0)	1 (33.3)
Rural, *n* (%)	22 (45.8)	11 (40.7)	6 (50.0)	5 (55.6)	–	1 (50.0)	2 (50.0)	2 (66.7)
Professional status								
Employees, *n* (%)	17 (35.4)	11 (40.7)	4 (33.3)	2 (22.2)	0.438	1 (50.0)	1 (25.0)	0 (0.0)
Homemakers, *n* (%)	12 (25.0)	8 (29.6)	3 (25.0)	1 (11.1)	–	0 (0.0)	1 (25.0)	0 (0.0)
Retired, *n* (%)	19 (39.6)	8 (29.6)	5 (41.7)	6 (66.7)	–	1 (50.0)	2 (50.0)	3 (100.0)
Anthropometric parameters								
BMI (kg/m^2^), mean ± SD	31.8 ± 1.7	31.5 ± 1.5	31.8 ± 1.7	32.2 ± 1.9	0.518	31.9 ± 1.6	32.0 ± 1.7	32.8 ± 2.1
Lifestyle factors								
Current smoking, *n* (%)	21 (43.8)	11 (40.7)	6 (50.0)	4 (44.4)	0.889	1 (50.0)	2 (50.0)	1 (33.3)
Alcohol consumption								
Alcohol intake (g ethanol/day), median (IQR) *	19.3 (14.2–19.7)	18.9 (2.8–19.7)	19.3 (18.9–19.7)	39.5 (19.7–39.5)	<0.001	19.7 (19.7–19.7)	39.5 (39.5–51.3)	29.6 (24.7–34.5)
Metabolic comorbidities								
Hypertension, *n* (%)	42 (87.5)	22 (81.5)	11 (91.7)	9 (100.0)	0.218	2 (100.0)	4 (100.0)	3 (100.0)
Diabetes mellitus/IFG, *n* (%)	24 (50.0)	12 (44.4)	7 (58.3)	5 (55.6)	0.684	1 (50.0)	2 (50.0)	2 (66.7)
Dyslipidemia, *n* (%)	37 (77.1)	20 (74.1)	9 (75.0)	8 (88.9)	0.612	2 (100.0)	3 (75.0)	3 (100.0)
Laboratory parameters								
Platelets (×10^3^/µL), mean ± SD	199.0 ± 58.4	214.6 ± 47.2	191.3 ± 53.9	152.8 ± 42.0	0.003	171.5 ± 34.2	149.3 ± 38.6	126.7 ± 29.8
GGT (U/L), median (IQR)	105.0 (51.8–215.5)	54.0 (44.5–105.5)	133.5 (94.8–206.0)	265.0 (225.0–337.0)	0.011	289.5 (277.2–301.8)	245.0 (222.2–283.0)	443.0 (280.0–836.5)
Total bilirubin (mg/dL), mean ± SD	1.57 ± 1.20	1.12 ± 0.62	1.64 ± 0.93	2.78 ± 1.41	0.002	1.98 ± 0.74	2.65 ± 0.91	3.92 ± 1.08
INR, mean ± SD	1.16 ± 0.42	1.03 ± 0.21	1.14 ± 0.33	1.52 ± 0.61	0.006	1.21 ± 0.19	1.46 ± 0.28	2.05 ± 0.37
Albumin (g/dL), mean ± SD	3.88 ± 0.60	4.12 ± 0.41	3.82 ± 0.44	3.29 ± 0.52	0.001	3.71 ± 0.32	3.28 ± 0.41	2.89 ± 0.36
Non-invasive fibrosis scores								
FIB-4 score, mean ± SD	1.74 ± 0.83	1.32 ± 0.56	1.76 ± 0.69	2.87 ± 0.91	<0.001	2.11 ± 0.42	2.79 ± 0.58	3.84 ± 0.67
APRI score, mean ± SD	0.99 ± 0.71	0.62 ± 0.41	0.96 ± 0.58	1.89 ± 0.83	<0.001	1.21 ± 0.39	1.90 ±0.25	3.42 ± 0.54
Cirrhosis-related clinical features (cirrhosis subgroup only)								
Ascites, *n* (%)	–	–	–	–	–	0 (0.0)	4 (100.0)	3 (100.0)
Hepatic encephalopathy, *n* (%)	–	–	–	–	–	0 (0.0)	1 (25.0)	3 (100.0)
Prior variceal bleeding and/or portal hypertension, *n* (%)	–	–	–	–	–	0 (0.0)	2 (50.0)	3 (100.0)
Transient elastography performed at baseline, *n* (%)	41 (85.4)	27 (100.0)	12 (100.0)	2 (22.2)	<0.001	2 (100.0)	0 (0.0)	0 (0.0)

Note: Continuous variables are expressed as mean ± SD or median (interquartile range), as appropriate. * Alcohol intake values are presented as median (interquartile range, IQR). An en dash (–) indicates that the *p*-value is not applicable. Alcohol intake was estimated from self-reported beverage type, volume, and frequency using standard alcohol content assumptions (beer 5%, wine 12%, spirits 40%) and an ethanol density of 0.789 g/mL. Only entries with clearly reported frequency were converted. Abbreviations: BMI, body mass index; GGT, gamma-glutamyl transferase; IFG, impaired fasting glucose; INR, international normalized ratio.

**Table 2:** Longitudinal evolution of clinical and non-invasive fibrosis markers according to alcohol abstinence status.

Parameter	Baseline (Total, *n* = 48)	Abstinent 6 Months (*n* = 35)	Abstinent 12 Months (*n* = 35)	Non-Abstinent 6 Months (*n* = 13)	Non-Abstinent 12 Months (*n* = 13)	*p* (Time × Group Interaction)
BMI (kg/m^2^)	31.7 ± 1.7	30.1 ± 4.2	29.6 ± 4.1	34.0 ± 5.1	34.3 ± 5.2	<0.01
GGT (U/L), median (IQR)	105.0 (51.8–215.5)	88.0 (64.0–112.0)	69.0 (48.0–86.0)	285.0 (220.0–360.0)	310.0 (250.0–395.0)	<0.001
FIB-4 score	1.74 ± 0.83	1.32 ± 0.61	1.18 ± 0.54	2.64 ± 0.93	2.82 ± 0.97	<0.001
APRI score	0.99 ± 0.71	0.72 ± 0.49	0.61 ± 0.43	1.58 ± 0.81	1.74 ± 0.88	<0.001
FibroScan (kPa)	8.9 ± 3.4	7.1 ± 2.6	6.4 ± 2.3	13.2 ± 4.1	14.1 ± 4.6	<0.001

Note: Data are expressed as mean ± SD or median (interquartile range), as appropriate. *p*-values were obtained using repeated-measures ANOVA testing the time × group (alcohol abstinence status) interaction; for non-normally distributed variables, the corresponding non-parametric alternative was applied, as appropriate. Abbreviations: BMI, body mass index; GGT, gamma-glutamyl transferase; FIB-4, fibrosis-4 index; APRI, Aspartate Aminotransferase-to-Platelet Ratio Index.

## Data Availability

The data supporting the findings of this study are not publicly available due to ethical and privacy restrictions, as they contain sensitive clinical information. De-identified data may be made available from the corresponding author upon reasonable request and with appropriate institutional approvals.

## Supplementary Materials

The following supporting information can be downloaded at: https://www.mdpi.com/article/10.3390/jcm15062257/s1, Table S1: Correlations between clinical and paraclinical parameters and non-invasive fibrosis scores at baseline; Table S2: Baseline non-invasive liver fibrosis scores according to type of alcohol-related liver disease; Table S3: Distribution of liver disease categories and comparative demographic, socio-professional, clinical and fibrosis characteristics at 6-month follow-up according to alcohol abstinence status; Table S4: Multivariate analysis of factors associated with liver fibrosis severity at baseline.

## Abbreviations

ALD	Alcohol-Related Liver Disease
ALT	Alanine Aminotransferase
ANOVA	Analysis of Variance
APRI	Aspartate Aminotransferase-to-Platelet Ratio Index
AST	Aspartate Aminotransferase
BMI	Body Mass Index
CAP	Controlled Attenuation Parameter
FIB-4	Fibrosis-4 Index
GGT	Gamma-Glutamyl Transferase
HBV	Hepatitis B Virus
HCV	Hepatitis C Virus
HDL-C	High-Density Lipoprotein Cholesterol
IFG	Impaired Fasting Glucose
INR	International Normalized Ratio
IQR	Interquartile Range
kPa	Kilopascal
MASLD	Metabolic Dysfunction–Associated Steatotic Liver Disease
MCV	Mean Corpuscular Volume
MetALD	Metabolic and Alcohol-Associated Liver Disease
NAFLD	Non-Alcoholic Fatty Liver Disease
NCEP ATP III	National Cholesterol Education Program Adult Treatment Panel III
OR	Odds Ratio
SD	Standard Deviation
