# Supplementary material for: Alcohol Abstinence Is Associated with Regression of Non-Invasive Fibrosis Markers in Patients with Metabolic Syndrome: A 12-Month Prospective Study
Source: J Clin Med. 2026 Mar 16;15(6):2257. doi: 10.3390/jcm15062257 (PMC13026559; doi:10.3390/jcm15062257)
Supplement: Supplementary file 1 [file jcm-15-02257-s001.zip › jcm-4138198-supplementary.pdf]

Journal of
Clinical Medicine

MDPI

Supplementary Table S1. Correlations between clinical and paraclinical parameters and non-invasive fibrosis scores at baseline.

Parameter	FIB-4 (r)	APRI (r)	FibroScan (r)	*p*-value
Age	0.61	0.54	0.58	<0.01
BMI	0.42	0.39	0.46	<0.01
AST	0.69	0.73	0.55	<0.001
ALT	0.58	0.61	0.49	<0.01
GGT	0.58	0.51	0.63	<0.01
Platelets	−0.62	−0.59	−0.57	<0.01
≥2 metabolic comorbidities	0.55	0.48	0.52	<0.01

Supplementary Table S2. Baseline non-invasive liver fibrosis scores according to type of alcohol-related liver disease.

Fibrosis parameter	Steatosis (*n* = 27)	Steatohepatitis (*n* = 12)	Cirrhosis (*n* = 9)	*p*-value
FIB-4 score, mean ± SD	1.32 ± 0.56	1.76 ± 0.69	2.87 ± 0.91	<0.001
APRI score, mean ± SD	0.62 ± 0.41	0.96 ± 0.58	1.89 ± 0.83	<0.001
FibroScan liver stiffness (kPa), mean ± SD	6.7 ± 2.1	9.4 ± 2.9	14.8 ± 4.3	<0.001
Fibrosis stage by FibroScan	F0–F1	F1–F2	F3–F4	-
Significant fibrosis (F ≥ 2), *n* (%)	4 (14.8)	6 (50.0)	7 (77.8)	<0.001
Advanced fibrosis (F ≥ 3), *n* (%)	1 (3.7)	3 (25.0)	6 (66.7)	<0.001

Supplementary Table S3. Distribution of liver disease categories and comparative demographic, socio-professional, clinical and fibrosis characteristics at 6-month follow-up according to alcohol abstinence status.

Characteristic	Abstinent (*n* = 35)	Non-abstinent (*n* = 13)	*p*-value
Type of liver disease at 6 months			
Steatosis, *n* (%)	22 (62.9)	5 (38.5)	0.048
Steatohepatitis, *n* (%)	9 (25.7)	3 (23.1)	0.842
Cirrhosis, *n* (%)	4 (11.4)	5 (38.5)	0.021
Demographic characteristics			
Age (years), mean ± SD	58.6 ± 7.9	69.8 ± 6.2	<0.001
Male sex, *n* (%)	24 (68.6)	8 (61.5)	0.735
Female sex, *n* (%)	11 (31.4)	5 (38.5)	–
Environment of origin			
Urban, *n* (%)	21 (60.0)	5 (38.5)	0.183
Rural, *n* (%)	14 (40.0)	8 (61.5)	–
Professional status			
Employees, *n* (%)	15 (42.9)	2 (15.4)	0.048
Homemakers, *n* (%)	10 (28.6)	2 (15.4)	–
Retired, *n* (%)	10 (28.6)	9 (69.2)	<0.01
Anthropometric parameters			
BMI (kg/m^2), mean ± SD	30.1 ± 4.2	34.0 ± 5.1	<0.05
Lifestyle factors			
Current smoking, *n* (%)	15 (42.9)	6 (46.2)	0.831
Metabolic comorbidities			
Hypertension, *n* (%)	29 (82.9)	13 (100.0)	0.171

Diabetes mellitus / IFG, *n* (%)	19 (54.3)	5 (38.5)	0.330
Dyslipidemia, *n* (%)	26 (74.3)	11 (84.6)	0.702
Non-invasive fibrosis scores at 6 months			
FIB-4 score, mean ± SD	1.32 ± 0.61	2.64 ± 0.93	<0.001
APRI score, mean ± SD	0.72 ± 0.49	1.58 ± 0.81	<0.001
FibroScan (kPa), mean ± SD	7.1 ± 2.6	13.2 ± 4.1	<0.001

Supplementary Table S4. Multivariate analysis of factors associated with liver fibrosis severity at baseline.

Predictor	FIB-4 (β, 95% CI)	*p*-value	APRI (β, 95% CI)	*p*-value	FibroScan F ≥ 2 OR (95% CI)	*p*-value	FibroScan F ≥ 3 OR (95% CI)	*p*-value
Age (years)	−0.002 (−0.019 to 0.015)	0.843	0.002 (−0.013 to 0.016)	0.838	0.87 (0.73–1.04)	0.124	1.05 (0.91–1.22)	0.487
Male sex (vs. female)	−0.059 (−0.487 to 0.369)	0.787	−0.111 (−0.460 to 0.238)	0.532	0.01 (0.00–2.56)	0.106	0.32 (0.02–5.94)	0.442
BMI (kg/m²)	0.018 (−0.119 to 0.155)	0.794	0.006 (−0.107 to 0.119)	0.916	0.82 (0.37–1.82)	0.623	1.14 (0.51–2.57)	0.749
log(1 + GGT)	0.552 (0.169 to 0.935)	0.005	0.459 (0.162 to 0.757)	0.002	140.6 (1.54–12,857.5)	0.032	5.81 (0.41–81.9)	0.195
Platelets (per 10^4/μL)	−0.036 (−0.085 to 0.014)	0.155	−0.036 (−0.076 to 0.003)	0.074	0.58 (0.30–1.12)	0.104	0.71 (0.35–1.46)	0.356
